# Lymphadenopathy Associated With the COVID-19 Vaccine

**DOI:** 10.7759/cureus.13524

**Published:** 2021-02-23

**Authors:** Nurith Hiller, Shraga Nahum Goldberg, Malena Cohen-Cymberknoh, Vladimir Vainstein, Natalia Simanovsky

**Affiliations:** 1 Radiology, Hadassah Hebrew University Medical Center, Jerusalem, ISR; 2 Paediatrics, Hadassah Hebrew University Medical Center, Jerusalem, ISR; 3 Hematology, Hadassah Hebrew University Medical Center, Jerusalem, ISR

**Keywords:** covid-19, covid-19 vaccine, regional lymphadenopathy, ultrasound, side effects

## Abstract

The coronavirus disease 2019 (COVID-19) pandemic has dominated nearly everyone’s life since its initial outbreak in the Hubei province of China in December 2019. The disease had spread quickly throughout the world causing extensive, widespread morbidity, over two million deaths, and economical and social devastation over the entire world. Researchers and pharmaceutical companies around the globe have been racing to develop potent and safe vaccines for the disease. Pfizer-BioNTech COVID-19 vaccine followed by Moderna COVID-19 mRNA-1273 vaccine were the first to receive FDA approval. These vaccines are based on messenger RNA novel technology and considered efficient in preventing contagion ensuring safety. Known side effects for this vaccine have been reported as very similar to those known for other vaccines. Specifically, lymphadenopathy has not been considered a common manifestation of COVID-19 vaccination.

Israel has been cited as leading in the introduction of these vaccines, which are available for every citizen older than 16 years.

Here, we present the cases of three patients who developed lymphadenopathy after the first dose of Pfizer-BioNTech COVID-19 vaccine. Time elapsed from the injection until the appearance of the enlarged nodes, clinical symptoms, and sonographic features differed between the patients, but in all cases gradual regression was noted in the enlarged nodes until complete resolution. Accordingly, to our knowledge, this is the first report describing post-COVID-19 vaccine lymphadenopathy detailing the clinical aspects, sonographic features, and outcomes.

## Introduction

The coronavirus disease 2019 (COVID-19) worldwide pandemic outbreak has engendered substantial research, manufacture, and use of messenger RNA (mRNA) vaccines. Although mRNA vaccine technology is relatively new compared to the long history of conventional vaccines to key pathogen proteins, it has been investigated during the last three decades on several viruses such as influenza, rabies, CMV (cytomegalovirus), and Zika [1-3]. COVID-19 mRNA vaccines currently approved for use by the FDA contain particles of mRNA that when injected to the body instruct our body’s cells to produce spike protein, a harmless surface protein unique to SARS-CoV-2, the COVID-19 virus. This spike protein is recognized by the body as an antigen provoking an immune response with the production of T-lymphocytes and B-lymphocytes targeted to destroy this specific antigen and allow for future immunity against the same pathogen. The Pfizer-BioNTech COVID-19 vaccine was the first to receive FDA approval. This vaccine is given as a set of two injections separated in time by at least 21 days. The most common reported side effects for the COVID-19 vaccine are similar to those reported in other vaccines in general, including pain in the injection site, fatigue, headaches, fever, chills, and muscle and joint pains. Lymphadenopathy has been reported as being rare [4]. Severe allergic reaction may occur but is very rare.

We present three cases of lymphadenopathy developed after Pfizer-BioNTech COVID-19 vaccination.

## Case presentation

Case 1

**Figure 1:**
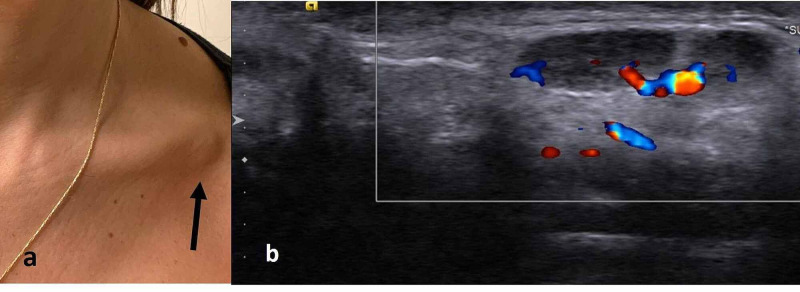
(a) Focal swelling in the left infraclavicular area that prompted the study (arrow). (b) Ultrasound image of the area demonstrating enlarged lymph nodes with a slightly hypoechoic cortical layer but with preserved hilum and blood supply.

A 47-year-old woman, generally in good health, presented to the Hematologic Clinic with a new non-painful left infraclavicular lump to rule out lymphoproliferative disease. Upon clinical examination, it was revealed that 15 days earlier she had received the first shot of the Pfizer-BioNTech COVID-19 vaccine. Shortly after the vaccination, she suffered from fatigue, myalgia, and mild pyrexia to 38°C. All these side effects resolved within 24 hours, and she was well ever since until she noticed an infraclavicular lump at day 15. Physical examination was unremarkable except for this small infraclavicular lump consistent with an enlarged node. The hematologist suspected that this may be associated with the vaccine, but because of the atypical location of the node she was referred for an ultrasound that was performed the day after. On Doppler ultrasound, slightly enlarged lymph nodes were noted in the infraclavicular area on the side of the vaccine injection, with preserved architecture and blood flow (Figure 1). Clinical follow-up was advised, and it was recommended that the second dose of the vaccine be given in the contralateral right arm. Lymphadenopathy resolved gradually until complete disappearance after 30 days. The second dose was given on the contralateral right arm, and the patient experienced slight pain at the injection site for several hours with no further side effects.

Case 2

**Figure 2:**
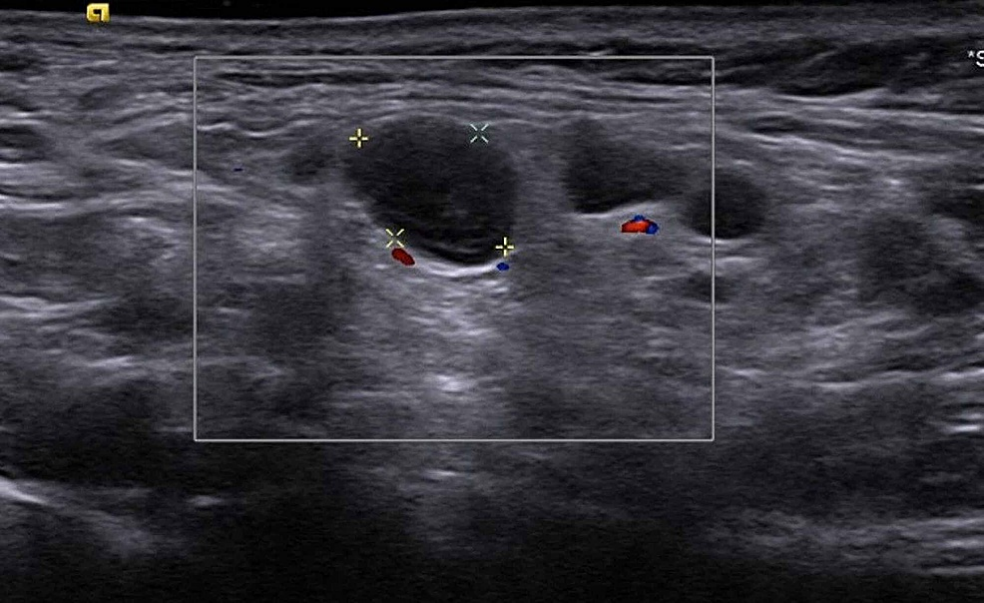
Ultrasound image of the supraclavicular area demonstrating hypoechoic rounded lymph nodes without identifiable hilum and without visible blood flow.

A 46-year-old healthy woman presented to the Hematologic Clinic with a new left supraclavicular and axillary painful lump. She received the first dose of the Pfizer-BioNTech COVID-19 vaccine five days prior to presentation. Immediately after the injection, she suffered from injection site pain and over the course of the next two days she experienced headaches, chills with normal body temperature, and a sense of “heaviness” of the neck and upper chest. These symptoms gradually resolved within two days. A painful lump appeared five days after the first injection after all other symptoms disappeared. Physical examination revealed a prominent painful lump palpated at the supraclavicular area as well as axillary area with local warmness. The rest of the examination was unremarkable. All blood tests including full chemistry, blood count, and CRP levels were within normal limits. On Doppler ultrasound examination performed on the same day, multiple enlarged lymph nodes were seen in the axillary, supraclavicular, and low lateral neck areas. These lymph nodes appeared homogeneously hypoechoic, without identifiable blood flow, consistent with necrotic changes (Figure 2). Pain over these lymph nodes as well as the size of the palpable lump gradually improved until complete resolution by day 23. The second dose of the vaccine was administered on day 21 from the previous one on the contralateral right arm. Eight hours post-injection, the patient suffered from severe arm pain causing marked difficulty in range of motion and chills followed by high fever that developed after 24 hours. These symptoms improved during the next two days until complete resolution at day 3 after the second vaccination. By day 4, painful axillary lymphadenopathy appeared and again demonstrated slow regression until complete resolution over the next two weeks.

Case 3

**Figure 3:**
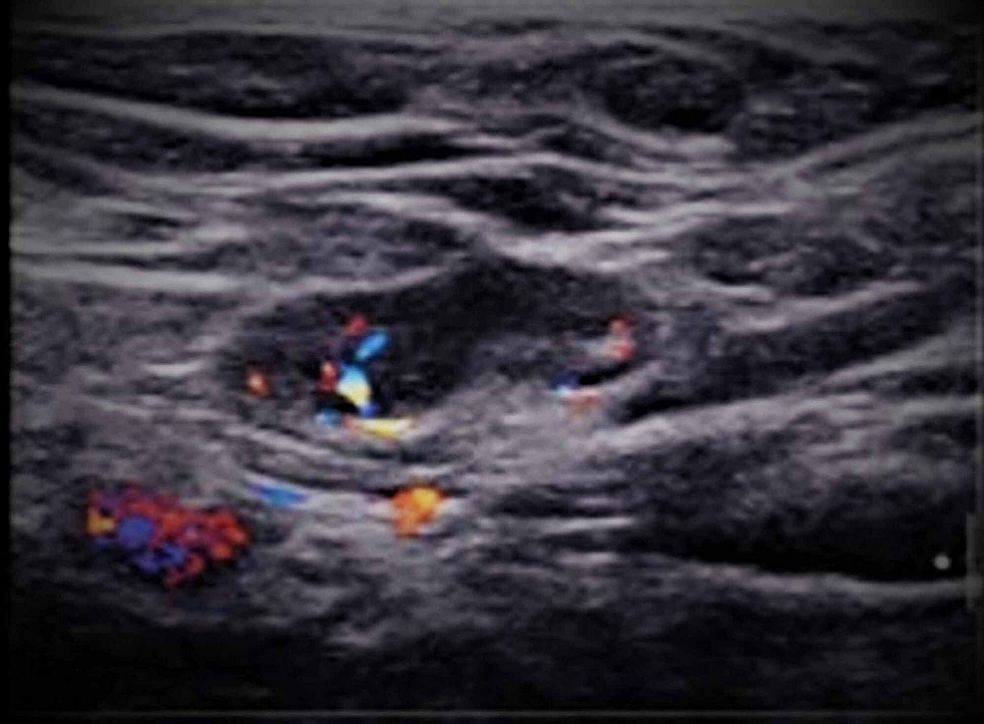
Ultrasound of the left axilla showing enlarged node with normal architecture and blood flow.

A 42-year-old healthy woman, BRCA gene carrier, presented to the hospital’s Breast Clinic for routine follow-up to rule out breast cancer. MRI revealed incidentally enlarged left axillary lymph nodes up to 2 cm in diameter. No suspicious finding was found in the left breast. Ultrasound of the left axilla showed several non-palpable and non-painful enlarged axillary nodes up to 2 cm in diameter with normal architecture and blood flow, suggesting a reactive nature (Figure 3).

**Figure 4:**
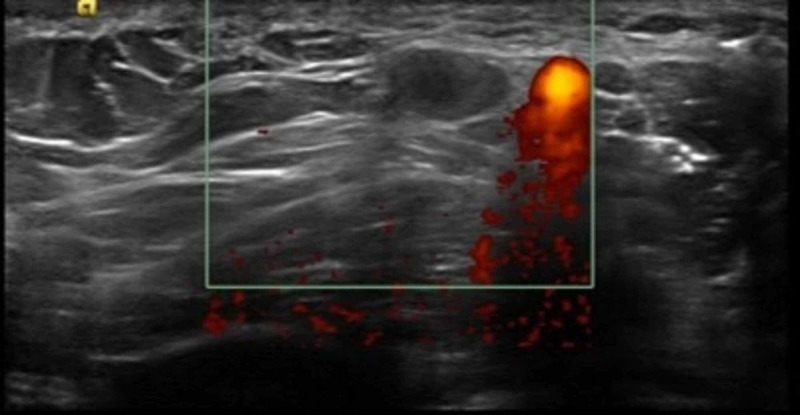
Ultrasonographic image demonstrating hypoechoic lymph nodes without blood flow, adjacent to the axillary vein.

Upon clinical examination, it was revealed that she had received the Pfizer-BioNTech COVID-19 vaccine to her left arm 18 days earlier with no obvious side effects except for mild soreness at the injection site. The patient received the second dose of the vaccine to the right arm three days later at day 21 as required, but she suffered marked side effects including severe arm pain, chills, diffuse myalgia, and fatigue. These symptoms improved gradually using NSAID (nonsteroidal anti-inflammatory drug) and disappeared after three days. In addition, approximately 24 hours from the second shot, a painful right axillary lump appeared, although it showed gradual improvement during the next week. Ultrasound of the right axilla was performed seven days after the second vaccine dose and demonstrated several enlarged nodes, most of them with preserved architecture and blood flow. The largest node measured up to 10 mm in diameter and was sensitive to local pressure, avascular with hypoechoic center, probably necrotic (Figure 4).

## Discussion

A gargantuan effort has been exerted in our country in an attempt to accelerate the vaccination of the entire population over a short period in order to contain the COVID-19 pandemic. With the widespread use of the new recently approved mRNA-based vaccines, we can appreciate the incidence and variety of the side effects associated with it. Similar reactions to self-remitting lymphadenopathy we report for the COVID-19 vaccine have been reported for several DNA or RNA-based vaccines (i.e., smallpox and include injection site pain and edema, fever, fatigue, myalgia, erythema, and lymphadenopathy) [5]. Regarding the Pfizer-BioNTech COVID-19 vaccine, lymphadenopathy is mentioned in the list of possible side effects, but it is not considered very common [4].

It is worth mentioning that we have described herein only three of many more cases of post-vaccination lymphadenopathy presenting to us within the first month of starting vaccination in our country. The number of cases was remarkable enough for us to report it.

Lymph node enlargement in our patients involved the supraclavicular, infraclavicular, and axillary regions on the same side of injection, consistent with lymphatic nodal levels draining the skin of the upper arm [6].

The appearance of new especially non-painful lymphadenopathy may be very disturbing. Family doctors, hematologists, breast surgeons, and radiologists must be aware of the fact that axillary, lower neck, supraclavicular, and infraclavicular lymphadenopathy may be related to the COVID-19 vaccine rather than representing actual more sinister pathologic processes. At the current time, patients presenting with lymphadenopathy must be asked whether they have received the vaccine recently.

In our series, ultrasound findings differed between painful and non-painful lymphadenopathy. Non-painful lymph nodes were enlarged, but normal architecture and blood flow were preserved. Painful lymphadenopathy was associated with loss of normal node anatomical landmarks, liquification, and avascularity consistent with node necrosis. Regardless of the clinical and sonographic appearance, all of the lymph nodes in our series showed gradual resolution within up to three weeks. Regardless, these cases show that lymphadenopathy may appear after the first and/or second dose of the vaccine, may be remote from the date of vaccine injection, and can be encountered after other more common side effects of the vaccine had resolved.

## Conclusions

In this era of COVID-19 pandemic and worldwide vaccination, medical staff should be aware that ipsilateral lymphadenopathy to the vaccine injection site may be a common side effect. COVID-19 vaccine related lymphadenopathy is associated with various clinical and sonographic features, but fortunately it shows spontaneous gradual recovery.
